# Between clinical practice, teaching and research – a project report on the development and implementation of a career mentoring curriculum for female clinician scientists

**DOI:** 10.3205/zma001556

**Published:** 2022-07-15

**Authors:** Christine Meyer-Frießem, Elena Enax-Krumova, Christiane Kruppa

**Affiliations:** 1 Ruhr-University Bochum, BG University Hospital Bergmannsheil gGmbH Bochum, Department of Anaesthesiology, Intensive Care and Pain Medicine, Bochum, Germany; 2 Ruhr-University Bochum, BG University Hospital Bergmannsheil gGmbH Bochum, Department of Anaesthesiology, Department of Neurology, Bochum, Germany; 3 Ruhr-University Bochum, BG University Hospital Bergmannsheil gGmbH Bochum, Department of General and Trauma Surgery, Bochum, Germany

**Keywords:** career mentoring, female clinician scientist, gender equality, curriculum development, postgraduate education

## Abstract

**Objectives:**

Despite the high proportion of female medical students, the leading positions in almost all medical departments are still considerably less often held by female clinicians than by male. The aim of this project report is to introduce a career mentoring curriculum for female clinician scientists at Ruhr-University of Bochum in Germany.

**Methods:**

The career mentoring program was developed for young female clinician scientists who aim for a leading position in academic medicine. We describe and discuss herein its planning and implementation over two years (11/2020-11/2022) focusing on the needs of the target group.

**Results:**

The mentoring program is based on an agile twin-track strategy. It includes peer mentoring, content-related input and interdisciplinary three-to-one mentoring by the coordinators. Additionally, the mentees develop and conduct a scientific project to practice their acquired skills in a protected environment. The quality assurance system of the mentoring program includes a continuous evaluation of the mentees’ satisfaction with the content and organisation of the program, whose results serve as the basis of the prompt adjustment of the latter. It also includes an evaluation of the sustainable impact of the program on the mentees’ career development based on an adapted role matrix. The role matrix addresses the mentees to acquire the competencies required for them to become a successful clinician, scientist and academic teacher.

**Conclusions:**

A 2-year innovative and unique career mentoring program for female clinician scientists was developed and already successfully launched. Making use of different mentoring strategies, the program has the potential to promote gender equality and encourage female physicians to pursue a career in academic medicine.

## Zusammenfassung

**Zielsetzung:**

Trotz des hohen Anteils weiblicher Medizinstudentinnen, sind die Führungspositionen in fast allen medizinischen Fachbereichen noch immer deutlich seltener von Klinikerinnen besetzt als von männlichen. Das Ziel dieses Projektberichts ist die Darstellung eines Karriere-Mentoring-Curriculums für klinisch und wissenschaftlich tätige Ärztinnen an der Ruhr-Universität Bochum in Deutschland.

**Methodik:**

Das Karriere-Mentoring-Programm wurde für junge klinisch und wissenschaftlich tätige Ärztinnen entwickelt, die eine Führungsposition in der akademischen Medizin anstreben. Die Planung und Implementierung des Programms über zwei Jahre (11/2020-11/2022), mit Fokus auf die Bedürfnisse der Zielgruppe, wird hier beschrieben und diskutiert.

**Ergebnisse:**

Das Mentoring-Programm basiert auf einer agilen zweigleisigen Strategie. Es beinhaltet Peer-Mentoring, inhaltlichen Input und interdisziplinäres Drei-zu-Eins-Mentoring durch die Koordinatorinnen. Darüber hinaus entwickeln und führen die Mentees ein wissenschaftliches Projekt durch, um ihre erworbenen Fähigkeiten in einem geschützten Umfeld zu erproben. Die Qualitätssicherung des Mentoringprogramms beinhaltet eine kontinuierliche Evaluation der Zufriedenheit der Mentees über den Inhalt und die Organisation des Programms, dessen Ergebnisse als Grundlage für eine prompte Anpassung des Programms dienen. Zusätzlich ist eine Evaluation der Nachhaltigkeit des Programms auf die Karriereentwicklung der Mentees auf Basis einer angepassten Rollenmatrix inkludiert. Die Rollenmatrix fokussiert auf den Erwerb der Kompetenzen, die benötigt werden, um eine erfolgreiche Klinikerin, Wissenschaftlerin und akademische Dozentin zu werden.

**Schlussfolgerung:**

Es wurde ein innovatives und einzigartiges zweijähriges Karriere-Mentoring-Programm für klinisch und wissenschaftlich tätige Ärztinnen entwickelt und bereits erfolgreich gestartet. Durch den Einsatz verschiedener Mentoring-Strategien hat das Programm das Potenzial, die Gleichstellung der Geschlechter zu fördern und Ärztinnen zu ermutigen, eine Karriere in der akademischen Medizin einzuschlagen.

## 1. Introduction

Mentoring originally belonged to the area of human resources development [1], although it has also been increasingly implemented in the scientific field, with established quality standards [2]. Mentoring programs have been shown to increase the mentees’ satisfaction and professional competencies [3]. In the last decade German medical faculties have introduced mentoring programs for both medical students [4], [5] and post-graduates, some of them focusing on women [6], [7]. Such programs aim to promote the hiring and retention of women in academic medicine [8]. The effectiveness of mentoring programs in enhancing diversity in academic medicine has been a contentious issue [9], but there have been a number of reports on their positive aspects (e.g. [9], [10]), and on German medical faculties’ positive experiences with them (e.g. [11], [12]). 

The mentoring programs that have been established at Ruhr-University Bochum in Germany have been designed addressing medical researchers in an interdisciplinary context (i.e. human medicine and natural sciences, basic and clinical science) [11]. These programs are also open to physicians, however, none of them focus on female clinician scientists. Young female physicians striving for a career as clinical scientists have specific needs that can be addressed only by a tailored mentoring program. Moreover, both basic researchers and clinicians benefit from interprofessional communication and networking, although their daily routines differ considerably. Physicians who pursue an academic career and aim to become a senior physician, or the head of a section or department have to simultaneously play three main professional roles: that of an academic teacher, that of a researcher, and that of a clinician involved in patient care. This can be challenging both in the early career stages and after a faculty position has been obtained [13]. 

In 2019, around two thirds of the students in human medicine were female. In contrast, the leading positions in almost all medical departments (with exception of the gynaecology and dermatology departments) are still considerably less often held by female clinicians than by male clinicians [14]. In 2019, the percentage of women in leading positions in clinical specialities (C4/W3 positions) in all the departments in German university hospitals was only 13% [15]. In terms of postdoctoral qualifications^1^ in human medicine/health sciences, the percentage of women was 35% [16]. These data refer to both human medicine and health sciences thus, the difference between habilitations and professorships cannot be directly calculated, but it is evident and far from parity. Thus, Ruhr University has established quantitative developmental goals to increase the proportion of women in permanent or temporary mid-level academic positions and junior professor staff positions [17]. Concerns about work–life balance, lack of adequate mentors and career role models, experiences of gender discrimination and unconscious bias in medical school and residency have been suggested as some of the reasons that many women do not want to pursue a career in academic medicine [18]. The lower scientific output of women in high-impact medical journals, their fewer citations in scientific papers [19], [20] and their lower levels of visibility in the society (as experts in the lay press) [21]] and in the scientific community [22], [23], [24] well manifest the current gender disparities in academic medicine. The poor visibility of women in the scientific community may also have an impact on the professional success of women themselves. Further, it results in less female role models. At the same time, the results of a review of the empirical evidence indicates that women’s participation in research can encourage women to pursue a career in academic medicine [18]. This could be achieved mainly by beginning research training early during residency or even already during medical school. 

To bridge the current aforementioned gap, the presented mentoring program aims to promote equal opportunities for young female physicians striving for a leadership position as a senior clinician scientist. To become at least a senior physician at a university hospital, it is essential but also challenging to acquire both profound clinical knowledge and research expertise. In the following, we describe the development process and implementation of this future-oriented project. Further, we discuss its future opportunities and possible limitations.

## 2. Program development

Driven by our own experience and the agreed-upon current objectives related to gender equality at Ruhr University Bochum, the program called *“MentÄ – Successful in clinics, academics and science”* was designed from April 2020 to November 2020. We followed the six-step approach for curriculum development in the context of medical education by Kern (Kern cycle), consisting of problem identification, target needs assessment, definition of goals and objectives, educational strategies, implementation and evaluation [25]. The knowledge and experiences from previous interdisciplinary mentoring programs at the Ruhr University [11] and elsewhere [3], [6], [18], [26] were adopted, focusing on the needs of young female physicians who aspire to obtain a position as a senior clinician scientist at a university hospital. Additionally, the program was substantially refined after needs assessment during the initial non-standardised interviews with the applicants.

The program has the following objectives:

- to encourage the program participants to engage in career thinking, and strategy development
- to motivate the program participants to consider career feasibility and to aspire for a leading position
- to encourage the program participants to engage in self-organisation and project management
- to encourage the participants to communicate and to make them visible
- to initiate and promote networking among the program participants and with others.

The Committee for Equal Opportunities of Ruhr University Bochum and the integrated Lore Agnes Program financially support gender-oriented programs every year [27]. The aforementioned 2-year mentoring program has been equally funded by the Lore Agnes Program and the Faculty of Medicine of Ruhr University Bochum.

### 2.1. Educational strategies

**Figure 1:**
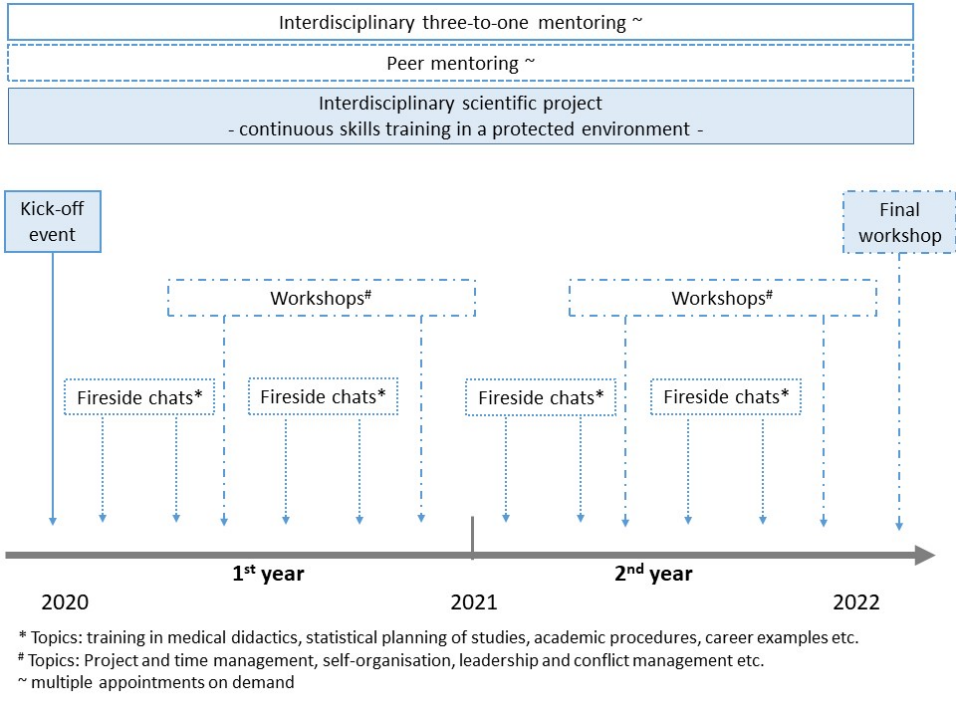
MentÄ – General concept and time line of the mentoring curriculum.

The mentoring program presented herein is based on a unique twin-track strategy. On the one hand, we adopted common features of other mentoring programs, such as peer mentoring, (modified) one-to-one mentoring and content-related workshops. On the other hand, we integrated an interdisciplinary project within the program to enable the program participants to practice all their acquired skills in a protected environment. Thus, the mentoring program includes the main blocks below (see figure 1 (Fig. 1)).

- common mentoring approach
  - **peer mentoring **
  - interdisciplinary **three-to-one mentoring** by the three coordinators
  - **content-related input** (i.e., three 1- to 1 ½-day workshops, 3-hour meetings every two months and fireside chats as often as requested)
- practical approach
  - development and implementation of an interdisciplinary **scientific project**

**Peer mentoring** is used to establish a network and to jointly strengthen the motivation for clinical research and teaching projects in addition to everyday clinical practice. The focus is on the participants (peers), who motivate each other in their individual careers. A short input on the topic of “collegial advice” at the beginning provides the theoretical basis for peer mentoring including an agreement on the use of discretion. Subsequently, in a group discussion, the participants regularly reflect on their goals and interests and receive feedback from the other peer group members on their work plan, with the support of the coordinators. The networking between the peers is intended to facilitate exchange within the scientific network and to motivate the participants to focus on career development with its various aspects at an early stage.

In addition, the mentees get individual feedback and input about strategic career planning from the coordinators **(three-to-one mentoring)**. Thus, the coordinators also serve as a “mentor team” with an interdisciplinary background from the surgical and conservative fields covering multiple facets of the work of a clinician scientist. Further, they continuously share their latest experiences on the balancing act between clinical practice, research, teaching and private life.

Another important component of the program is the **content-related workshops** offered to strengthen competencies for future clinical research and to give input on possibilities and important milestones for a clinical career. These include self-organisation, project management, leadership, communication skills and conflict management. 

Simultaneously, the mentees develop their own mutual **scientific project**. Thus, they are being encouraged to actively participate in academic medicine [18] and to practice project management skills in a protected environment through jointly strengthened motivation with possibilities of new areas of cooperation. In the interdisciplinary project the mentees are accompanied by the three coordinators, who provide support with regard to content-related and organisational issues.

The concept of the program is based on agile planning, for the implementation of peer mentoring and networking in a pandemic situation with contact restrictions and a high workload burden in hospital staff. 

#### 2.2. Organization of the program

The executive team of the 2-year program consists of three female clinicians acting as both coordinators and mentors, each representing a different clinical discipline (anaesthesiology, neurology and trauma surgery). The program, including the research project, is accompanied by a student assistant for administrative tasks (6 hours per week over 2 years). In contrast to most other medical faculties, the university hospitals of the Medical Faculty of Ruhr University Bochum are located at multiple different sites (“Bochum model”). The program equally addressed female physicians from all of these sites.

#### 2.3. Participants

To obtain the best possible benefit from such a mentoring program, the candidates were required to be at least in the second year of their residency and to possess prior research experience (e.g. having successfully written a medical dissertation). For application purposes, a curriculum vitae and a motivation letter describing the candidate’s previous scientific, academic teaching and clinical experience and their career development goals was required. The candidates were formally interviewed in detail regarding their motivation for applying in the program, their individual personal and career development needs and their specific needs depending on their specialty. A group size of six mentees was intended to allow intensive interaction. 

#### 2.4. Quality assurance

**Figure 2:**
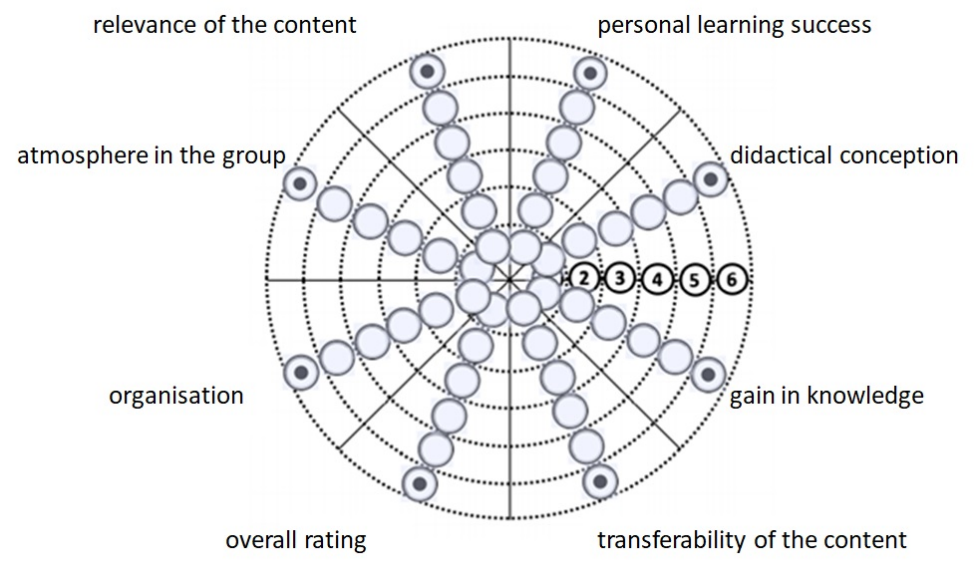
Target for evaluation of each meeting, adapted from [28]. With one point in each segment of the evaluation target, the mentees should rate every session or meeting regarding their satisfaction with the eight indicated subareas. Based on this target tool they can quickly answer the question “Am I satisfied with ...?” referring to the eight areas based on a six-point Likert scale ranging from 1 (middle of the target, “very satisfied”) to 6 (most outer dotted ring, “not satisfied at all”). The closer all points are set to the middle, the more positive is the evaluation.

During the program all meetings with content-related input (workshops, 3-hour meetings and fireside chat) have to be formally evaluated by each mentee in terms of their satisfaction with the meeting organisation, didactical conception, presentation, individual knowledge gain, personal learning success, climate within the group and content relevance and transferability. The answers to the question “Am I satisfied with ...?” are based on a six-point Likert scale ranging from 1 (“very satisfied”) to 6 (“not satisfied at all”), displayed visually as a target diagram (see figure 2 (Fig. 2), adapted from [28]). The evaluation and the ongoing improvement efforts enable a continuous adjustment of the program based on the mentees’ needs.

Further, we used a role matrix for the mentees’ individual reflection on their personal development and visualisation of the program success. We applied a competence-oriented role matrix that was adapted from the curriculum of the Master of Medical Education program in Germany [29] and that ensures high-quality standards [30]. It was initially conceived as an outcomes-based framework (CanMEDs) of physician competencies (i.e. roles) for all areas of medical practice (medical expert, professional, communicator, scholar, collaborator, leader and health advocate) [31]. The role matrix has recently been shown to be suitable for objectively evaluating the results of a mentoring program [32]. We adapted it to the concept of the present mentoring program and focused on the three roles that the mentees have to play for a successful career in academic medicine: clinician, academic teacher and researcher. Each role has three levels in which the mentees are active/visible in their professional community: department/team, hospital/faculty and national/international. We also differentiated between three competence levels (collaboration/practice under supervision, self-determined performance and leadership/developmental function). The mentees were asked to extensively reflect on their roles and current levels shortly before the kick-off meeting. At the end of the program (2 years from its start), a further reflection of the individual roles and accomplished goals is planned to be performed. In addition, a re-assessment is intended 3 years after the completion of the mentoring program.

## 3. Results

### 3.1. Implementation

The program was announced university wide. Due to the so-called “Bochum model” of the Medical Faculty of Ruhr University Bochum, we addressed university hospitals across different cities in North Rhine-Westphalia. Owing to the large number of motivated and qualified applicants (three times more than the vacant places), the number of participants was increased from six to eight. The female physicians were in their second to fifth year of residency in surgical or conservative specialties from five different university hospitals of Ruhr University Bochum. This diversity was intentional for building the basis for peer mentoring and for addressing different aspects of the mentees’ career development. All the applicants voluntarily applied for the program, and the directors of their departments approved their program participation.

Due to the current COVID-19 pandemic situation with contact restrictions, however, the program was implemented in a hybrid fashion as it was known that a mentoring relationship is personal and has to develop in a protected space. The kick-off event took place in November 2020 with a strict hygienic concept, thus allowing the peer mentoring group members to initially personally get in touch with each other. The further meetings took place, however, through video calls. Here, all technical opportunities (e.g. breakout sessions) were used to allow interactive personal exchanges and discussions of different issues both in plenum and in small groups. Mentoring programs with online formats have already been proven to be feasible [33], but the extent to which they can replace in-person mentoring meetings remains unclear. Therefore, the future workshops and meetings were planned to take place either through video conferencing or in person, depending on the pandemic situation.

The kick-off meeting included a content-based input from the coordinators about the goals and mentoring approach of the program and about their clinical, scientific and personal background. Further, the mentees introduced themselves to the whole peer group and in small groups, describing their clinical, scientific and personal backgrounds and their current and future career goals, based on their individual role matrices. 

#### 3.2. Components

##### Workshops, meetings and fireside chats

To advance the development of non-technical soft skills [34], workshops, networking meetings and fireside chats were integrated into the mentoring program. The program coordinators did not define the content of these up front, however, as they focused on the individual needs of the mentees. Possible topics were assessed, discussed and ranked together with the mentees to decide on the foci of the future mentoring sessions. The topics that were consented to include self-management, project management, communication skills and conflict management, scientific writing, statistics, acquisition of teaching skills, how to acquire research support from third-party funding, leadership skills and others. The mentees further requested that they be made to meet role models and learn about the individual experiences and career strategies of these. For this, fireside chats with female and male professors and senior scientists were organised. 

##### Peer mentoring and three-to-one mentoring

Peer mentoring as a valuable tool for advancing soft skills and mind sets [35], took place regularly and on demand within the group of eight participants, with or without the attendance of the three coordinators. Additionally, the three coordinators offered regular and on-demand personal exchanges and mentoring with each participant to extensively discuss individual issues with the participant and to make them reflect on such issues. The topics included work-life balance challenges for female clinicians, self-organisation, visibility and others [36].

##### Interdisciplinary scientific project

In a second block of meetings, the mentees were motivated to develop an interdisciplinary scientific project and to accomplish it within the 2-year mentoring period, giving the mentees an opportunity to practice project management and collaborative work in a protected environment. To find a common research interest among the mentees and to identify their individual knowledge and resources, the mentees were made to share their experiences and interests. Possible study designs and themes were discussed in the peer groups after literature research to define a concrete research topic. A project plan was designed, and different tasks were distributed among the mentees, with assistance by the coordinators. Deadlines for milestones were set, and the progress of the project was tracked. For this purpose, a team software was provided during the mentoring program, which could be used for networking between the participants and the coordinators, for the participants’ self-management and for the management of the interdisciplinary research project.

The average time spent on all the activities was estimated to be 2 hours per week for the mentees and 1 hour for each of the coordinators during the 2-year period, exclusive of the student assistance.

## 4. Discussion

The presented unique mentoring program for female clinician scientists at Ruhr University Bochum is based on a twin-track approach, focuses on the balancing act between clinical, research and academic engagement and private life and aims to promote equal career opportunities.

Although the project has the potential to be valuable, it has some a priori limitations in terms of both implementing the introduced components and evaluating their effectiveness for the professional development of the mentees. First, despite the predefined inclusion criteria for unifying the members of the peer group to a certain degree, there was a natural inhomogeneity and variability between the mentees regarding their curriculum vitae, individual aims, professional qualifications and medical disciplines. Previous knowledge, experience and skills but also professional and private environment differed between the mentees, at least partly. Therefore, mentees might benefit to different extent from the mentoring program. Second, the influence of external (positive or negative) factors such as professional environment, family, friends, general health and socioeconomic factors can hardly be entirely addressed during the mentoring process. Nevertheless, addressing all individual influencing factors of mentoring programs on the career is rather a limitation in general. Third, evaluation of the project’s success is crucial. However, it can be assessed indirectly, and comparison to a control group is not feasible due to the selection bias in favour of those who are motivated to participate in a mentoring program, for example, with a tendency to already have clearer professional aims in the early career stage. 

Until now, the effectiveness of mentoring in reducing gender inequalities has been controversially discussed [9]. Mentoring seems to be capable of improving female academic mentees’ job-related well-being, self-esteem and self-efficacy already within 6 months, with further improvements after 1 year [10], [11]. Nevertheless, there are external factors within the mentees’ institutional environment and private context that cannot be addressed. Firstly, most program evaluations have been survey based, with participant-reported satisfaction being the most frequently measured outcome [8]. Thus, the success of the program itself can hardly be proven due to a probable continuous evolution of the mentees. In particular, it has to be assumed that the mentees are already highly interested and engaged actors. Secondly, unfortunately, such interventions can currently address only a small number of mentees. 

It is worth noting that the proposed program offers a unique opportunity to likewise focus on the requirements within clinical settings, research, academic engagement and private life. Linked by a twin-track strategy, theoretical and practical knowledge can be simultaneously obtained in a protected environment. Additionally, the project benefits from network mentoring by several interdisciplinary mentors domiciled in the disciplines of anaesthesiology, neurology and surgery. Including heterogenous personalities in a peer group can also reach both the shy mentees and the crowd pullers at the same time. The interaction between the residents of different medical specialties enables different perspectives on clinical daily routines, research approaches and career paths in academic medicine. Further, organising such a multidimensional project enables networking not only at one’s own department and specialty but also within the entire medical faculty, including all the university hospitals within the “Bochum model”. With fireside chats, interdisciplinary mentoring by the coordinators, workshops and a mutual research project, the program addresses important reasons that many women do not pursue careers in academic medicine [18].

Even though the meetings, workshops and fireside chats are regularly being evaluated and the role matrices are regularly being reflected on within the peer group, at this point of the ongoing program, measurement of the program’s success is not possible. In addition, further mentee groups will have to conduct the program and the evaluations need to be performed also 5 or 10 years after the program. 

For the future, individual career planning needs to be started early, such as already during high school [37] or during medical studies [38], and such programs must be carried out routinely during residency and in the postdoc phase to encourage recruitment of under-represented minority groups for leading positions in academic medicine. Furthermore, it is essential to promote intensive support on a much broader basis. Multidimensional tools (e.g. electives, workshops, one-to-one or peer mentoring) could allow the broad masses to be addressed, already motivating female students during their medical studies. One example is the elective subject “Career Management for Medical Students” connecting the topics of gender sensitivity and career management through a short-term mentorship among medical students in the ninth semester at Leipzig University [26]. Another mentoring program has been offered at the University of Hamburg for all the students in the second semester of medical school, but with further focus within specific modules on the top 10% of the students with excellent achievements and also on the 10% with the most study difficulties [5]. At the start of the implementation of the *“MentÄ – Successful in clinics, academics and science”* mentoring program the authors already established an elective clinical course named *“Career pathways for future female clinicians”* without any precedent at Ruhr University to enable the students to come in contact with the concepts of equality, female leadership and career planning early in their medical studies [39].

Whereas the proposed project focuses on gender equality, future career development programs should address not only women. Similar projects should address all underprivileged participants, whenever needed, to prevent unfair paths and conflicts [40]. In fact, the programs’ goal should be the provision of equal opportunities for all motivated and qualified actors. 

## 5. Conclusion

An innovative and unique 2-year career mentoring program for female clinicians (*“MentÄ – Successful in clinics, academics and science”*) was developed and successfully launched at Ruhr University Bochum. Making use of different mentoring strategies, the program has the potential to promote equal opportunities for female clinical scientists and encourage young female physicians to be an active part of the academic medicine.

## Note

^1^ The postdoctoral qualification or habilitation is the highest qualification of a university career in Germany and other European countries. It qualifies to teach in a university and to obtain a professorship.

## Author contributions

All three authors contributed equally.

## Acknowledgement

First, we thank the *“Lore-Agnes-Programm”* and the Medical Faculty of Ruhr-University Bochum for financial support of the described mentoring program. Second, we thank our mentees for their trust and engagement. We also thank Helga Rudack for consulting us during the various stages of the program and Alina Funhoff for her support as medical student assistant within this project. Finally, we thank our heads of departments and the hospital management for their support leaving room for individual preferences. 

EEK holds an endowed professorship funded by the German Social Accident Insurance (DGUV) for the time of 6 years (2020-2026).

## Competing interests

The authors declare that they have no competing interests.
